# Visual Attention in Real Classrooms: A Study with Eye-Tracking in Urban and Rural Schools of Chile

**DOI:** 10.3390/jemr19020032

**Published:** 2026-03-18

**Authors:** Marco Villalta-Paucar, Jéssica Verónica Rebolledo-Etchepare

**Affiliations:** Escuela de Psicología, Universidad de Santiago de Chile, Santiago 9170022, Chile; jessica.rebolledo@usach.cl

**Keywords:** eye-tracking, cognitive development, primary education, classroom, rural, urban, visual attention

## Abstract

Student gaze behavior has been scarcely studied in real Latin American primary school classrooms. The objective of this study is to analyze the relationship between primary students’ eye behavior and cognitive development in urban and rural contexts. A quantitative method was employed, including 126 primary school students aged 6 to 8 years old, from urban and rural schools in Chile. Raven’s Colored Progressive Matrices (CPM) measured cognitive development, and students’ eye behavior was recorded during a real class using eye-tracking glasses. Eye behavior was analyzed in six areas of interest: (1) Own material (2) teacher, (3) teacher’s material, (4) peer, (5) peer’s material, and (6) non-interactional gaze. The results indicate that the CPM scale demonstrates adequate reliability (α = 0.89). In addition, no significant differences, nor relationship between eye behavior and cognitive development, were found by sex; however, significant differences were found by environment (urban versus rural). The regression analysis is significant (F(7, 102) = 6.173, *p* < 0.001) and suggests that gazing at the teacher’s material and one’s own material are negative predictors of non-interactional gaze or students’ disconnection from the class. In conclusion, distraction in the classroom is influenced by learning-related contextual variables rather than sex or cognitive development.

## 1. Introduction

Paying attention is the first cognitive step toward learning and is directly related to academic performance [1,2,3]. Capturing primary students’ attention represents both a challenge and a fundamental task for teachers, and is one of the aspects that define their classroom management expertise [4].

In the classroom, attention is an individual behavior that coordinates with the actions of the teacher and peers. The coupling of primary students’ interbrain attention—measured through electroencephalogram (EEG)—with the common class dynamic is positively correlated with academic performance [1]. Likewise, the systematic review of studies using eye-tracking with students reveals that the gaze is a relevant indicator of attention at school, e.g., in reading comprehension and mathematic cognition activities [5,6,7,8].

Specifically, the eye behavior of primary school children is different due to developmental factors, as found by comparing typical development to that of children on the autism spectrum (ASD) [6], the monocular fixation of the gaze becomes more stable as age increases [9], and some socioeducational factors, such as teaching activities based on active rather than traditional learning, increase the time students gaze at the materials presented by the teacher in the classroom [10]. Both development and learning build cognitive development [11]; in this sense, the educational process in the classroom does not only contribute to the learning of academic knowledge but also promotes the cognitive development of students. The use of eye-tracking in primary education can enhance interactivity, keep students engaged, and provide teachers with immediate feedback [12]. Portable eye-tracking devices are also a feasible option for sports tests with young children to evaluate their motor skill development [13], given the connection between motor and ocular functions in school-age children [14]. Students who experience difficulties with reading fluency tend to spend more time fixating on words and exhibit faster eye movement while reading [15]. Additionally, eye-tracking enables the capture of detailed data on students’ mathematical thinking processes and of non-verbal communication [16]. In addition, the analysis of students’ eye behavior opens up the possibility to explore the relationship between visual processes and cognitive development [8].

Instruments for assessing the cognitive development of primary school students from Latin America are varied and measure skills such as intelligence, language, and non-verbal reasoning [17]. In primary education, one of the most widely validated tests is the Raven’s Colored Progressive Matrices (CPM), as it assesses the cognitive functions of attention and self-regulation, independent of students’ linguistic development, which makes this test suitable for use in small children who have not developed verbal language yet [17].

The CPM has been used to establish the relationship between cognitive skills and the intake of specific nutrients [18,19] to establish the relationship between cognitive development and academic performance [2], between cognitive development and neurodevelopmental factors [20,21], and between CPM and eye behavior in amyotrophic lateral sclerosis (ALS) patients with severe physical disability, finding that neuropsychological tests based on eye-tracking are efficient to assess cognitive deficits in patients who cannot write or speak, like patients with severe ALS [22]. Furthermore, a recent study links the CPM test with eye behavior and physical growth, indicating a positive association between this scale and height by age, as well as an inconsistent association between CPM and eye behavior [23].

In school contexts, studies based on Vygotsky’s cultural–historical psychology use CPM for the dynamic assessment of proximal cognitive development after educational interventions that promote learning autonomy [24,25] in diverse sociocultural contexts [26,27]. Self-awareness when responding to Raven’s colored test has been found to improve students’ performance [28].

Eye behavior, in particular eye fixations, can provide valuable and reliable information for classifying processes and cognitive load [29], as well as guidelines for the design of educational material with enough visual stimuli and that does not overload the working memory of students [30,31]. Different visual activity patterns have been reported among young men and women [32]. Likewise, there are significant differences in the behavior of young adults and fourth grade children when reading a text [33]. Cultural differences and professional experience also influence the gaze patterns of teachers [34]. However, educational interventions that improve understanding, such as irony, are not necessarily associated with specific gaze behavior [35]. Therefore, further research is required in this field.

Systematic reviews on research using eye-tracking in education report an increase in its application between 2021 and 2023 compared with previous years, being especially predominant in the European environment and among university students [5,7] and extensively applied in the fields of cognitive science and education technologies [36]. In addition, reviews focused on the field of mathematical education indicate that, since 2023, studies have been based on preschool, primary, and secondary students, underscoring the use of static eye-tracking tools in systems based on desktops or screens [37]. Nevertheless, studies on gaze conducted in real classroom settings, spaces where the role of gaze is ecologically validated in relation to teaching and learning, are scarce [38].

Studies on the gaze behavior of teachers in real classrooms have reported cultural and contextual differences in classroom management [34,39,40] and have contributed to the discussion about teaching practice [41,42]. No studies were found that employ eye-tracking tools through devices mounted on the head, such as glasses, with primary students in real classrooms of Latin America. In this region, social inequalities, especially in urban and rural sectors, are related to educational, mental health problems, and implications in cognitive development [43,44], which creates a heterogeneous reality in the classrooms.

In summary, research on eye behavior in education has examined its relationship with cognitive development, school performance, and the optimization of teaching practices. However, most of these studies have been conducted in controlled settings, which deliberately reduce essential contextual factors—such as noise, class size, or the diversity of didactic activities—that influence the attention and susceptibility to distraction of students. Consequently, these artificial conditions do not always capture the real dynamics and daily interactions between teachers and students. The complex reality of the classroom thus challenges the stability of relationships observed in controlled environments. Moreover, there is a clear lack of research in rural classrooms, with most studies conducted in controlled and urban contexts.

The analysis of the interaction between gaze, attention/distraction, and cognitive development can provide valuable insights into the role of context in eye behavior and its implications for learning. Consequently, this study offers a novel contribution by examining this relationship from the students’ perspective within the real-word contexts of Latin American classrooms.

Therefore, in the context of real and heterogeneous primary education classrooms, the research questions are: what is the behavior of students’ gaze? What relationship can be established between gaze and cognitive development? This study aims to analyze the relationship between the eye behavior of primary students in the classroom and their cognitive development in urban and rural contexts. The study focuses on the gaze of students who use pupil-tracking glasses, introducing into the classroom context an advanced technological tool that allows for analyzing visual attention in real time.

## 2. Materials and Methods

### 2.1. Participants

The sample consisted of 126 first and second grade primary students aged 5 to 8 years, comprising 56 students from rural classrooms (25 boys and 31 girls) and 70 students from urban classrooms (35 boys and 35 girls) from Chilean schools located in urban (Metropolitan region) and rural (La Araucanía region) areas. Data were collected between 2015 and 2018. According to data from the government of Chile [45], the 19 schools participating have an educational vulnerability index (IVE) ranging from medium to high. This index is calculated based on the income of the family, housing conditions, and access to health and recreation services, among other indicators. The children from rural schools in this study have the highest educational vulnerability indexes in Chile.

### 2.2. Instruments

Raven’s Colored Progressive Matrices Scale (CPM) measures the development of the cognitive skills of comparing and reasoning by analogy, solving problems through abstract reasoning, and without depending on verbal language in children [46]). The test is on a booklet that contains 36 problems depicted incompletely in colored drawings, with alternatives provided to complete the figure and a single one as the right one. The test is organized in three series: A, Ab, and B, with 12 drawings of increasing complexity each, from the easiest, series A, to the hardest, series B. The test is applied in studies about overall intelligence in the fields of clinical practice and education in diverse contexts and languages [28]. The reliability of the CPM has been studied in Latin American culture and specifically in Chile [47,48].

For wireless eye-tracking glasses, the Tobii Pro Glasses 2 (Tobii Technology K. K., Tokyo, Japan) model was employed, an eye-tracking system equipped with four eye-tracking cameras—two per eye—that capture pupil movement and eye orientation, along with a simplified calibration process focused on one point, which can be completed in a few seconds. The device features a front-facing camera with 1920 × 1080-pixel resolution, four infrared sensors for pupil detection and tracking, and an integrated microphone for audio recording. The gaze sampling rate is 100 Hz. Glasses are connected to a recording unit installed in the participant’s pocket. Students put on eye-tracking glasses at the beginning of the class. Accuracy of the wireless recording was ensured through calibration, i.e., adjusting the eye model of each participant to the scene recorded by the camera before class. In addition, the percentage of gaze samples captured by each record is reported by the Pro v.1.6.7 software.

A SONY HDR-CX440 (Sony Corporation, Minato-ku, Tokyo, Japan) recorder in a fixed position was used for the complementary recording of the interaction events in the classroom. The fixed camera was placed at the bottom of the classroom, facing the whiteboard and behind the students.

### 2.3. Procedure

The research process was adjusted to ethical protocols approved by the ethics committee of research accredited in Chile. The research team informed management teams, teachers, and guardians about the objectives and procedures of the study. Informed consent and assent letters were signed prior to the collection of classroom recordings.

The first activity was the application of the CPM collectively in the classroom during school time, following the protocols indicated by the author [48]. Subsequently, data collection dates and times for classroom recording were coordinated with schools, teachers, and students’ guardians. Class recording was conducted 5 to 10 days after the application of the CPM. Both activities were carried out between 2015 and 2018. Two children used eye-tracking glasses in each class. Classes lasted 45 to 60 min. Teachers chose the students using the glasses, considering their willingness as well as adapted and integrated behavior during class activities as selection criteria. Some students removed their glasses during class. Such records were not included in the study. Ninety-six classes were filmed, obtaining 126 recordings.

The curricular activities performed in class corresponded mostly to the Spanish Language Arts, which has the objective of teaching reading and writing, but activities from other subjects such as Natural Sciences and Mathematics were also included. The teachers chose the activity for which they allowed recording. Recordings were conducted during real, full-length lessons, with their duration and content aligned with the teaching schedule and school calendar. Students participated with their usual classmates. For this study, only two children per classroom wore Tobii Pro Glasses 2 glasses. Considering that the installation of a single classroom camera and the use of eye-tracking glasses by only two students could elicit general curiosity and affect natural class dynamics, a rehearsal session, or ‘session 0’, was conducted. During this session, the entire setup process was carried out without recording data, allowing students to become accustomed to the presence of the recording equipment and research team. In addition, when students’ curiosity disrupted teaching, an extra measure was implemented: similar glasses without gaze-tracking functionality were given to the remaining students. This approach helped standardize the group’s perception of observation, reducing reactive behavior and controlling the curiosity factor. This was the case in some rural and urban classrooms, regardless of group size or specific activity, highlighting the variability of classroom management strategies employed by the teachers.

### 2.4. Data Analysis

The analysis of the CPM was conducted using the total score obtained, and the reliability of the instrument was calculated. To analyze students’ eye behavior, 1260 min of eye data were recorded using the Tobii Pro Glasses 2. In total, 126 10-min sessions were collected. To control observation time, the first 10 min of the class development phase were considered. This phase is defined as the period during which teachers present and implement activities to achieve the lesson objectives, which include detailed presentation of the objective, the presentation of videos, promotion of group discussions, and similar tasks.

Eye fixation was analyzed through the Tobii I-VT Attention algorithm. Its settings are predefined and optimized for portable eye-trackers used in glasses [49], making it well-suited for the study and allowing for quantifying the distribution of visual attention and the duration of fixations in the total sample of classes, corresponding to the stage of development, when the teacher conducted teaching activities. Eye behavior was recorded through tracking gaze attention fixation with the 1.171 Tobii Glasses Analyzer software, on a map of a static area of interest. The delimitation of areas of interest (AOIs) was based on the literature review on the role of gaze in diverse educational contexts, including communicative interaction between the teacher and students [12,50], the practice of mental math activities [8], the use of educational materials (such as reading or multimedia resources) [36,51], and peer interaction. In this way, six AOIs (Table 1) were established. One of these, ‘non-interactional gaze’, is defined as eye fixation on areas unrelated to the class activity. The application of this AOI definition for the manual recording of gaze fixation—measured in milliseconds and mapped on the static AOI layout—was conducted by two previously trained coders. To ensure data consistency and reliability, only 10 min recordings per participant that were approved by both coders were included in the analysis.

The Tobii Glasses Analyzer 1.171 software was used to record eye fixation on AOIs, generating results in units of seconds on an Excel spreadsheet. Subsequently, these files were imported to the SPSS v28 software for the corresponding statistical analysis.

Data analysis included the calculation of the main descriptive statistics, such as mean and standard deviation, to exhaustively characterize the sample and synthetize the central properties of the study variables: cognitive development and gaze. To ensure psychometric robustness, the reliability of the CPM was assessed through Cronbach’s alpha, a statistical technique used to determine the internal consistency of a construct, which is an essential methodological requirement for further analysis. As the eye behavior and cognitive development study variables were conceptualized and operationalized as continuous variables, the Pearson (r) correlation coefficient was employed to examine the bivariate associations between them. In addition, a comparison of the variable means was conducted based on sex and urban/rural area of participants using the Student’s *t*-test.

Finally, hypotheses were verified before conducting linear regressions, which included the inspection of Pearson’s correlation matrix to assess linearity, the analysis of variance inflation factors (VIFs) to detect multicollinearity, and the exam of the residual plot to analyze normality and homoscedasticity. This process aimed to explore the predictive capacity between the areas of interest of gaze, age, and the CPM.

## 3. Results

The CPM showed good internal consistency for the 126 study cases, with a Cronbach’s alpha α = 0.89 for the 36 scale items.

To ensure the reliability of the eye measurements, cases with a gaze capture percentage (gaze sample) below 60% were omitted. This threshold is considered optimal for research in natural classroom contexts, where the mobility of individuals and dynamic interactions can affect tracking continuity, achieving a balance between sample representativeness and the precision of the data analyzed. As a result, 110 cases (Table 2) were used for subsequent analysis.

Table 2 shows that gaze fixation was mostly directed to the teacher and the material presented in class (45.4%) compared to fixation on student material, peers, and their material (28.6%). This difference may be related to the class dynamics and the recording stage, which was centered on the teacher.

The observed correlations were low; however, some were statistically significant (Table 3). A weak but significant correlation was found (r = 0.206, *p* = 0.03) between age and cognitive development measured by the CPM. There was no significant relationship between the areas of interest of gaze and the CPM.

The highest and most significant correlation occurred between gaze fixation on a peer and their material (r = 0.635, *p* < 0.001), i.e., students who looked at their classmates also tended to gaze at the material they were using. In addition, students who gazed at their own material (r = 0.367, *p* < 0.001) tended to pay less attention to the teacher (r = −0.271, *p* < 0.001) and the material they presented (r = −0.216, *p* = 0.02) and to display less non-interactional gaze (r = −0.258, *p* = 0.007). In turn, non-interactional gaze was positively correlated with gazing at the teacher (r = 0.245, *p* = 0.01), which could be related to a tendency to get distracted during teacher instruction.

No significant differences were observed in gaze behavior or the CPM results when comparing them against the sex of participants (Table 4). The sex of participants did not influence their cognitive development or eye behavior patterns in the classroom.

**Figure 1:**
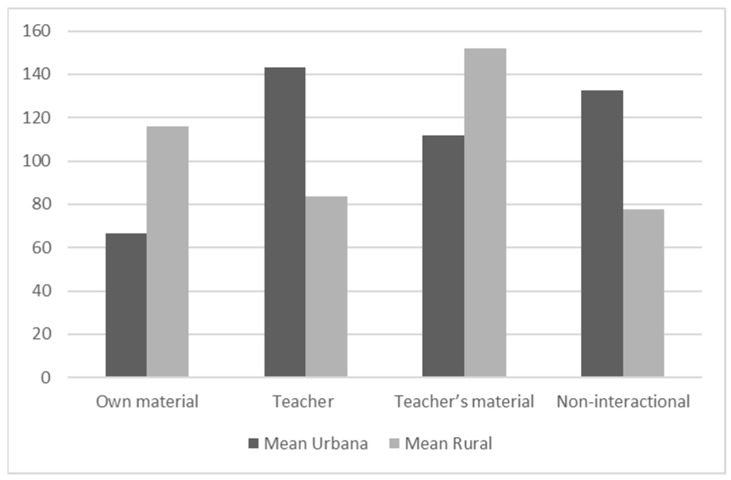
Comparison of urban and rural gaze means in statistically significant AOI differences.

Significant differences were found between urban and rural classrooms in most of the study variables (Table 5). The results of the Student’s *t*-test show that students from urban classrooms tended to gaze more at the teacher (M = 143.12; SD = 94.86) compared with rural students (M = 83.82; SD = 57.81), with a considerable effect size (Cohen’s d = 0.73). This group also presented, comparatively, a higher frequency of distraction (M = 132.59; SD = 69.58; *p* < 0.001; Cohen’s d = 0.84). In turn, students from rural classrooms dedicated more time to gazing at their work materials (M = 116.14; SD = 103.84; *p* = 0.01; Cohen’s d = −0.52) and the teacher’s material (M = 152.14; SD = 101.21; *p* = 0.04; Cohen’s d = −0.39), which suggests a learning dynamic focused on interaction with the class material (Figure 1). There were no statistically significant differences in cognitive development in the rural or urban context (*p* = 0.20) according to the CPM.

Based on correlation data and mean comparison, the cause-and-effect relationships between variables of cognitive factors, age, and the gaze of students in the classroom were explored. With this purpose, the residual independence, linearity, and multicollinearity hypotheses were tested, obtaining suitable tolerance values for the variance inflation factor (VIF < 2).

In the multiple regression model, non-interactional gaze was considered a dependent variable, while age, the CPM, gaze at peer’s material, gaze at own material, gaze at teacher, and gaze at teacher’s material were defined as predictor variables (Table 6). The model was statistically significant (F(7, 102) = 6.173, *p* < 0.001) and explained 29.8% of variance (R2 = 0.298). Two significant predictors were identified: gaze at own material (β = −0.503, *p* < 0.001) and gaze at teacher’s material (β = −0.351, *p* < 0.001), both inversely related. The other variables did not show significant influence. In sum, the reduction of gazing at one’s own material and at the teacher’s material contributed to non-interactional gaze.

In summary, data indicate that the cognitive process measured by the CPM is not a significant predictor of non-interactional gaze or student distraction. The reduction in gazing at one’s own material and teacher material accounted for 29.8% of the variance in non-interactional gaze. The significant correlation between gazing at one’s own material, peer material, and peers suggests that these variables are mutually reinforcing, reflecting a self-contained world within the classroom. Therefore, reduced gazing at one’s own material and teacher material predicts non-interactional gaze or student disconnection from the class.

## 4. Discussion

This study explored the relationship between the gaze of students in real classes and cognitive development via the CPM, using portable eye-tracking devices. One of its results points to the absence of a relationship between gaze behavior in the classroom and cognitive development. This confirms the findings of a similar study conducted with static eye-tracking in 10-year-old children, in which gaze patterns in written texts are not modified despite improvements in reading comprehension [35]. This study reveals that analogical reasoning and visual-spatial analysis—competencies independent of verbal language (Spanish) and assessed using CPM—are not related to students’ eye behavior in real classroom settings. Age was also found to be a better predictor than the results of the CPM, compared to the saccadic eye movement registered through eye-tracking [23].

Likewise, in this study, there were no statistically significant differences between boys and girls in the cognitive development scale, nor between the eye fixation time at the areas of interest in the real class—gaze at teacher, at own material, at peer, at peer’s material, at teacher’s material, and non-interactional gaze. The study by Cuesta-Cambra et al. [32] reported differences by gender but between young men and women, using static eye-tracking to assess a health education program.

The results of this study reveal that students’ attention, which manifests through their gaze behavior, is not conditioned by structural factors, such as sex and cognitive development. In real primary education classrooms of Chile, the attention of students is observed to be more influenced by the urban and rural conditions of the educational environments. In rural classrooms, where the number of students ranges between 5 and 10, children direct their gaze to their material and the teacher’s material more often. This environment is characterized by active use of worksheets that students complete assisted by the teacher, who circulates among the desks, promoting direct and personalized interaction. In contrast, in urban classrooms, with an enrolment of 30 to 40 students, teachers tend to adopt an expository approach, positioning themselves in the front part of the classroom. In these latter classes, not only is a more pronounced attention to the teacher observed but there is also a predominance of non-interactional gaze, in which students may be distracted or disconnected from learning.

These attention patterns can be explained by the cultural role of gaze in communication. In rural contexts, communication dynamics promote a more personalized interaction through a more individualized use of didactic material. This is in stark contrast with urban classrooms, where the higher number of students and the expository teaching approach generate an environment that fosters both attention to the teacher and focus dispersion manifested through non-interactional gaze. Therefore, these eye behaviors not only reflect the conditions of the classroom but also the ways of communication prevailing in each context, suggesting that the physical and social environment significantly impact the way students get involved in their own learning.

The role of culture in classroom behavior has been demonstrated in studies with higher education teachers from different cultures [34]. The novelty of this study lies in showing that the cultural differences and contextual conditions of the classroom are related to an inverse distribution of classroom gaze between primary education students of urban and rural classrooms. This contributes to a better understanding of the contextual and sociocultural nature of gaze behavior in real classrooms, specifically in the Latin American context.

Likewise, the results show that students’ attention, manifested in their classroom gaze behavior, constitutes a network of relationships between gazing at their own material, at peers, and at peers’ material. It is not only about “paying attention to the teacher”. However, there is a microcosm within the classroom where classroom management tends to promote that students inhibit their interest in their own material and that of their peers to focus on the teacher’s instruction. This finding shifts the focus from individual students to a pedagogy that takes advantage of the interaction among students as a way to potentiate learning.

It is often assumed that “more visual attention equates to better cognitive performance”. The findings indicate that, in real school performance contexts, gaze does not predict cognitive development. Several studies only focus on the student–screen or student–teacher dyad. Conversely, this study reveals a “distinct world” of peer dynamics, highlighting student areas of interest (AOIs) in the classroom, with a strong emphasis on interaction with their classmates and their materials. A horizontal attention ecosystem is observed between peers, which competes with the vertical gaze directed at teachers and their material.

Students use their gaze not only to interact with the teacher but also to self-regulate by looking at their own material, as well as to learn socially through gazing at their peers and their material. This “different world” of visual attention suggests that this is not purely individual action but rather it is deeply rooted in interpersonal dynamics and the management of available educational resources in the learning environment.

This study presents several limitations. First, the gaze records were conducted with 60% calibration, which means that some attentional gaze fixations may not have been captured. In addition, a real primary education class is structured as a didactic sequence that comprises the stages of lesson initiation, development, and closure. In this study, only the first 10 min of the development phase were recorded, which might have influenced some communication dynamics in terms of classroom management for 6-to-8-year-old students—these aspects were not addressed in this work. Another limiting factor is the type of class dynamic and the subject selected by the teachers, which may have impacted gaze behavior. The teachers’ experience in classroom management, as well as the adaptations made to gaze recording to accommodate the conditions inherent in a real classroom, are factors that could influence the studied relationships. Despite these limitations, this study pioneers in the use of eye-tracking with “glasses” in real and heterogeneous classrooms of Latin American primary education. The study addresses an important methodological and geographic gap, providing data with high ecological validity in a context marked by specific social and educational inequality [43,44]. This study reveals that, while cognitive development is not correlated with students’ gaze patterns, the rural or urban context of educational institutions is. This indicates that contextual or pedagogical factors—such as class size, didactic resources used, teaching methodology, and even the interaction dynamic promoted by the teacher—significantly influence students’ eye behavior. This perspective shifts the focus from a purely individual model to one that integrates environment and interaction.

In summary, this study provides a novel methodological contribution by being among the few studies to use mobile eye-tracking in natural primary school environments, thereby overcoming the low ecological validity that characterizes traditional laboratory- or screen-based research. This approach allowed us to observe the visual attention of young children (6–8 years old) in a real and dynamic classroom setting, a population and context that are both particularly challenging and rarely studied. In addition, the study offers a significant conceptual contribution by reexamining the predictors of attention in the classroom. While the literature often emphasizes individual cognitive development, the findings reveal that non-verbal cognitive abilities (measured by CPM) do not predict disconnection, suggesting that attention is a complex phenomenon mediated by multiple dynamic factors. Lastly, this study does not only address a methodological gap in the application of eye-tracking but also offers new theoretical and empirical insights into the multifactorial nature of attention in the classroom, with direct implications for pedagogy and for understanding socioeducational inequalities that emerge in daily interactions.

## 5. Conclusions

This study—a pioneer in the application of mobile eye-tracking technology in Latin-American primary school classrooms that considers urban–rural differences—reveals key factors underlying the nature of students’ attention.

The results confirm that eye behavior in the classroom is neither determined by structural factors such as sex nor associated with individual non-verbal cognitive development, as measured by CPM. This disassociation underscores the need for caution when interpreting visual attention as a direct and exclusive indicator of cognitive capacity, challenging purely individualistic perspectives.

In contrast, the learning environment appears to play a decisive role in attention. Significant differences were observed in the gaze patterns of students in urban and rural schools, indicating that contextual and pedagogical factors—such as class size, didactic resources used, teaching methods, and type of interaction promoted by teachers—significantly influence students’ visual attention. Specifically, while in urban classrooms students tended to gaze more toward the teacher and were distracted more frequently, in rural classrooms gaze was primarily directed toward the student’s own material and the teacher’s material, which suggests a different learning style.

These findings emphasize the intrinsically dynamic and socially mediated nature of visual attention, which constitutes a complex network of interaction between students, their classmates, and class materials. Educational interventions are, therefore, more likely to be effective when focused on teaching innovation and the optimization of resource presentation rather than exclusively on individual cognitive profile.

From a practical perspective, this study provides valuable data for curricular design and the implementation of didactic strategies for maximizing the learning experience, especially in heterogeneous contexts. Theoretically, this study frames attention in the classroom as a construct sensitive to ecological and socioeducational factors. Future research should include larger samples in rural and remote areas to confirm the generalizability of these findings and to deepen the understanding of the contextual specificities that shape attention in the classroom.

## Figures and Tables

**Table 1:** Definitions of areas of interest (AOIs) for the analysis of student gaze.

Areas of Interest	Definition
Own material	Gaze is fixed on the notebook, pencil, or material used by the students, as well as on their own hands when these are used as a resource, for example, the mathematical calculation of a sum.
Teacher’s material	Gaze is fixed on the whiteboard—when there is class material to pay attention to—notebook, or book used by the teacher in class.
Teacher	Gaze is fixed on the teacher’s face or hands. Frontal view of the teacher’s upper body, or when the teacher has their back turned while talking to the class.
Peer	Gaze is fixed on the student’s face, heads, or hands. The upper part of the body is in front of the user wearing eye-tracking glasses.
Peer’s material	Gaze is fixed on the notebook, pencil, or work material used by the student’s classmate.
Non-interactional gaze	Gaze fixation does not cover the categories above. The student looks at the ceiling, the wall, window, floor, door, or back of other students. It implies disconnection from the visual attention related to class activities.

**Table 2:** Descriptive statistics of the study variables.

	Mean (Years)	Mean (Score)	Mean (Seconds)	Standard Deviation	N
Age	6.72			0.65	110
CPM		21.85		6.49	110
Peer’s material			16.94	28.38	110
Peer			37.30	41.42	110
Own material			88.12	98.24	110
Non-interactional gaze			108.55	70.80	110
Teacher			117.25	85.73	110
Teacher’s material			129.29	105.37	110

**Table 3:** Pearson’s correlation for the gaze behavior of students by age and CPM scale.

	Age	CPM	Peer’s Material	Peer	Own Material	Non-Interactional	Teacher	Teacher’s Material
Age	1							
CPM	0.206 *	1						
Peer’s material	−0.139	−0.016	1					
Peer	−0.166	−0.005	0.635 **	1				
Own material	−0.118	−0.059	0.367 **	0.089	1			
Non-interactional	0.088	−0.056	−0.258 **	−0.095	−0.419 **	1		
Teacher	0.194 *	0.101	−0.271 **	−0.175	−0.524 **	0.245 **	1	
Teacher’s material	−0.025	0.068	−0.216 *	−0.044	−0.412 **	−0.112	0.165	1

* The correlation is significant at the 0.05 level (bilateral). ** The correlation is significant at the 0.01 level (bilateral). These correlations are placed in bold

**Table 4:** Comparison of means using Student’s *t*-test and effect size for the study variables by sex.

	Boys				Girls				t	df	*p*	d
Variables	N	M1	M2	SD	N	M1	M2	SD				
CPM	55	22.11		6.08	55	21.60		6.92	0.410	108	0.68	0.08
Peer	55		38.22	46.63	55		36.38	35.87	0.231	108	0.82	0.04
Own material	55		92.59	92.63	55		83.65	104.22	0.475	108	0.64	0.09
Teacher	55		114.89	80.33	55		119.60	91.50	−0.287	108	0.77	−0.05
Teacher’s material	55		125.24	105.23	55		133.34	106.31	−0.402	108	0.69	−0.08
Peer’s material	55		17.66	30.95	55		16.22	25.83	0.264	108	0.79	0.05
Non-interactional	55		109.96	70.59	55		107.14	71.63	0.208	108	0.84	0.04

Note: N = number of participants; M1 = mean (score); M2 = mean (seconds); SD = standard deviation; t = t-statistic; df = degrees of freedom; *p* = *p*-value; d = Cohen’s d.

**Table 5:** Comparison of means using Student’s *t*-test and effect size for the study variables by area (urban vs. rural).

	Urban				Rural				t	df	*p*	d
Variables	n	M1	M2	SD	n	M1	M2	SD				
CPM	62	22.55		6.37	48	20.96		6.60	1.279	108	0.20	0.25
Peer	62		40.24	44.73	48		33.50	36.82	0.846	108	0.40	0.16
Own material	62		66.42	88.55	48		116.14	103.84	−2.708	108	**0.01**	−0.52
Teacher	62		143.12	94.86	48		83.82	57.81	3.815	108	**<0.001**	0.73
Teacher’s material	62		111.60	105.91	48		152.14	101.21	−2.029	108	**0.04**	−0.39
Peer’s material	62		15.96	29.80	48		18.20	26.70	−0.408	108	0.68	−0.08
Non-interactional	62		132.59	69.58	48		77.50	59.96	4.370	108	**<0.001**	0.84

Note: n = number of participants; M1 = mean (score); M2 = mean (seconds); SD = standard deviation; t = t-statistic; df = degrees of freedom; *p* = *p*-value; d = Cohen’s d.

**Table 6:** Multiple linear regression analysis to predict non-interactional behavior.

Variable	B	SE	β	T	*p*
(Constant)	179.039	66.180	----	2.705	0.008
Gaze at peer’s material	−0.446	0.294	−0.179	−1.516	0.133
Gaze at teacher	0.002	0.082	0.003	0.030	0.976
Gaze at teacher’s material	−0.236	0.062	−0.351	−3.812	<0.001
Gaze at peer	0.086	0.190	0.051	0.455	0.650
Gaze at own material	−0.363	0.080	−0.503	−4.537	<0.001
Age	1.856	9.484	0.017	0.196	0.845
CPM	−0.752	0.931	−0.069	−0.808	0.421

Note. N = 110; R2 = 0.298; R2 adjusted = 0.249; F(7, 102) = 6.173, *p* < 0.001; B = non-standardized coefficient; SE = standard error; β = standardized coefficient.

## Data Availability

The data presented in this study are available upon request from the corresponding author to protect the confidentiality of the participants.
